# Bullous lesions as a manifestation of systemic lupus erythematosus in two Mexican teenagers

**DOI:** 10.1186/1546-0096-8-19

**Published:** 2010-07-08

**Authors:** Marimar Sáez-de-Ocariz, Francisco Espinosa-Rosales, Eduardo López-Corella, Beatriz de León-Bojorge

**Affiliations:** 1 Department of Dermatology. National Institute of Pediatrics, Mexico City, Mexico; 2 Department of Primary Immunodeficiencies Research, National Institute of Pediatrics, Mexico City, Mexico; 3 Department of Pathology. National Institute of Pediatrics, Mexico City, Mexico

## Abstract

Rarely, systemic lupus erythematosus (SLE) presents with bullous lesions due to severe edema and hydropic degeneration of the basal layer, or as a subepidermal blistering disease. Here, we describe two Mexican teenagers, one with SLE with blisters and another with bullous SLE. We also discuss the mechanisms and clinical implications of lesion formation in patients with SLE and bullae.

## Background

Bullous lesions can occur in systemic lupus erythematosus (SLE) as a subepidermal blistering disease [1] or when severe edema and hydropic degeneration occur in the basal layer. The latter condition is considered a lupus erythematosus (LE)-specific lesion [2]. The former condition is a rare disorder characterized by tense fluid-filled vesicles and bulla, with an erythematous or urticarial background. This condition is very rare in children, with only eleven cases reported to date [3-13] (Table 1). Here, we describe two females, aged 13 and 16 years, who presented with bullous lesions during the course of SLE.

**Table 1:** Pediatric Cases of Bulllous SLE

Author & Year	Sex	Age	Clinical features	Histopathological features
Jacoby & Abraham 1979^3^	Male	15 years	Multiple tense vesicles and bullae, arising on normal skin on the face, chest, axilla and inner arm	Subepidermal blister with neutrophils, eosinophils and lymphocytes.
				DIF^¥ ^IgG depositis in the BMZ^£ ^under and around the vesicle

Hall et al 1982^4^	Male	12 years	Small vesicles on an erythematous base on arms, face, chest and legs.	Subepidermal bullae with neutrophilic microabscesses in dermal papillary tips.
				DIF: linear IgG, IgA, IgM and IgE and granular C3 deposits at the dermo-epidermal junction.

Kettler et al 1988^5^	Female	8 years	Bullous tense eruption over the face, hairline, neck, axilla, groin, ankles, palms and soles.	Subepidermal bullae with neutrophilic infiltrate as well as papillary microabscesses adjacent to the bulla.
				DIF: Granular IgG and C3 deposits at the dermo-epidermal junction

Roholt et al 1995^6^	Female	9 years	Linear papulovesicular eruption over the right dorsal hand and ulnar forearm.	Subepidermal split, mixed cellular infiltrate with neutrophils.
				DIF: strong IgG, IgA and IgM linear deposition at the dermo-epidermal junction

Shirhama et al 1998^7^	Female	9 years	Widespread blisters on the trunk, neck, and oral mucosa.	Subepidermal blister with a neutrophilic infiltrate in the upper dermis.
				DIF: linear IgG, IgA and C3 deposition at the BMZ.

Chan et al 1999^8^	Female	15 years	Numerous tense bulla on her entire skin surface	Subepidermal blister, neutrophilic infiltrate in papillary dermis and blister cavity.
				DIF: IgG, IgA and C3 deposits at the BMZ.

Nitta Y et al 2002^9^	Male	17 years	Disseminated blistering eruption	Papillary microabscesses of neutrophils and subepidermal blisters.
				DIF: IgG, IgA, IgM and C3 deposits at the BMZ.

Harris-Stith et al 2003^10^	Female	17 years	Photodistributed tense bulla on the face (lips and ears) and upper torso, as well as oral and vaginal erosions	Neutrophil-rich subepidermal bullae.
				DIF: IgG, IgM and C3 deposits at the dermo-epidermal junction

Sirka et al 2005^11^	Female	18 years	Vesicles on the left retroauricular area, left upper eyelid, right axilla, chest and abdomen.	Subepidermal bullae with neutrophilic microabscesses.
				No DIF was performed.

Vijayalkshmi & Jayavardhana 2007^12^	Male	10 years	Vesicles and bulla on ears, neck, shoulders, arms and legs	Subepidermal blister with neutrophilic dermal infiltration.
				DIF: strong IgG and moderately strong IgM, IgA and C3 granular BMZ deposition.

Kumar &Agarwal^13^	Female	13 years	Numerous bulla and vesicles on the back, abdomen, neck, flexures of the arm and groin.	Dermo-epidermal separation and a neutrophil-predominant inflammatory infiltrate in the upper dermis.
				DIF: Prominent fluorescence along the epidermal basement membrane for IgG, IgA, IgM and C3.

¥ DIF = Direct Immunofluorescence; £ BMZ= Basal membrane zone

## Report of cases

### Case 1

**Figure 1:**
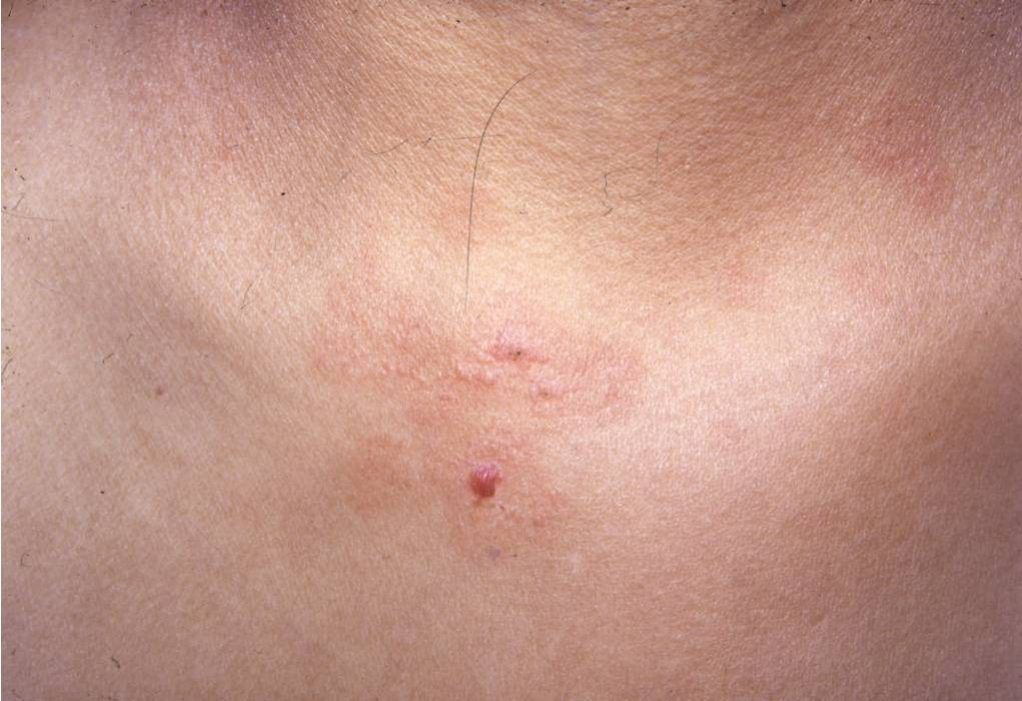
**Several 1 mm to 2 mm clear and hemorrhagic tense vesicles were observed over erythematous macules on the patient's neck**.

A 13-year-old female presented with a macular and vesicular eruption of 3 days duration. On examination, several 1 mm to 2 mm clear or hemorrhagic tense vesicles over erythematous macules were identified in the left supracilliary region and on the neck (Figure 1). Several 10 mm to 20 mm erythematous macules were also present on the patient's trunk. Both the vesicles and the macules were pruriginous.

The patient had been diagnosed with SLE and lupus nephritis 2 months prior to the onset of the dermatosis, based on hemolytic anemia, lymphopenia, hypocomplementemia [C4 8 mg/dL (15-45), C3 116 mg/dL (83-177)], proteinuria (2.3 g/day), and positive antinuclear antibodies (1:640, diffuse pattern). She also had positive anti-dsDNA antibodies, positive anti-Ro antibodies, positive low titer IgM (26 MPL) and IgG (21GPL) anticardiolipin antibodies. She was prescribed oral prednisone and received three methylprednisolone boluses. The current dermatosis coincided with a new episode of hemolytic anemia and resolved with slight depigmentation within 10 days, after a fourth methylprednisolone pulse.

**Figure 2:**
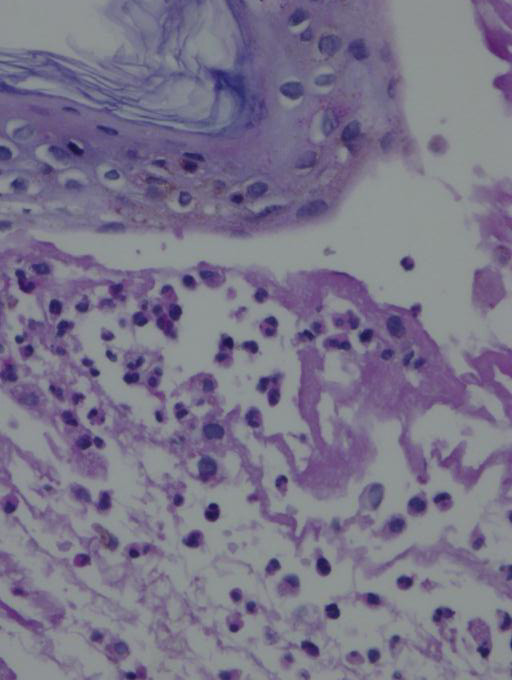
**A subepidermal blister with marked neutrophilic dermal infiltration**.

Histopathological study of a skin biopsy revealed a subepidermal blister with marked neutrophilic dermal infiltration (Figure 2) and a slight perivascular neutrophilic infiltrate. Direct immunofluorescence showed strong granular basement membrane zone IgG, IgA, C1q, and C3 deposition, and moderate granular vessel IgA and C1q deposits.

The patient did not experience another bullous eruption in the 3 years following that episode. Her SLE has predominantly been characterized by nephritis and hematologic disease, which have gradually resolved with cyclophosphamide, methylprednisolone boluses, and oral prednisone. During her most recent follow-up exam, cytotoxic treatment was suspended, oral prednisone was being tapered and hydroxychloroquine treatment had commenced.

### Case 2

**Figure 3:**
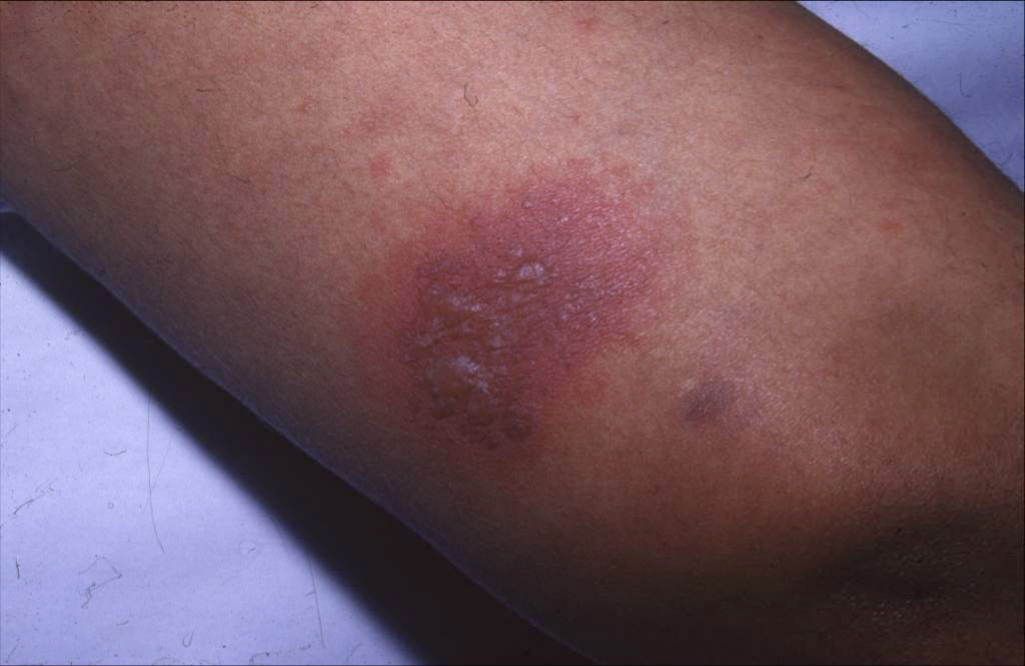
**A purpuric plaque with clear-fluid blisters on the surface**.

A 16-year-old female presented with a purpuric and bullous eruption of 7 days duration. On examination, several 3 cm to 6 cm nummular purpuric plaques were disseminated on her trunk and extremities. The surface of each plaque contained a clear-fluid blister (Figure 3).

The patient had been diagnosed with SLE and secondary antiphospholipid syndrome (APS) 1 year prior to the onset of the dermatosis, based on hemolytic autoimmune anemia, lymphopenia, thrombocytopenia, proteinuria, serositis, cutaneous vasculitis, positive antinuclear antibodies, positive antiphospholipid antibodies, and a thrombus on the inferior cava vein. During the year prior to the onset of the bullous dermatosis, the patient had experienced several disease flares associated with persistent lymphopenia, with the main target organs being the central nervous system (vasculitis), kidney and liver. She was treated with methylprednisolone and cyclophosphamide boluses, as well as warfarin for APS, topiramate for seizures, and captopril for high blood pressure. During this period, the patient also developed warfarin skin necrosis and experienced three different septic events (due to *E. faecium*, *Candida sp *and *Aspergillus*) that required aggressive antimicrobial treatment and the replacement of warfarin with low-dose aspirin.

**Figure 4:**
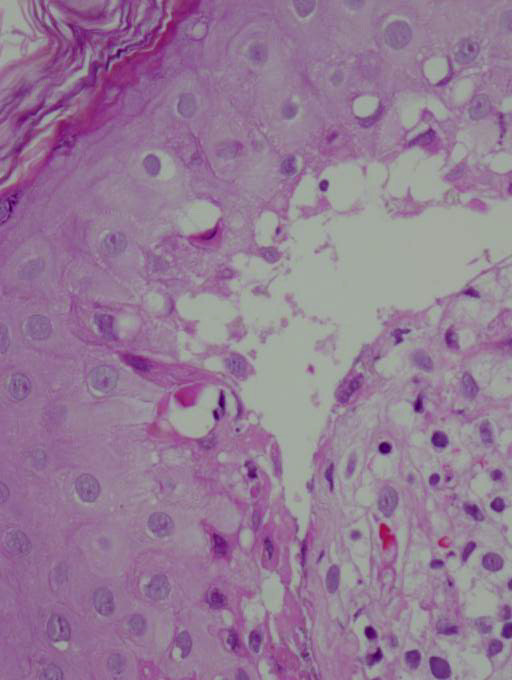
**Epidermis with keratinocyte necrosis, severe basal liquefaction degeneration with blister formation and papillary dermal edema**.

Histopathological examination of the bullous lesions revealed an atrophic epidermis with marked keratinocyte necrosis, as well as severe hydropic degeneration of the basal layer with blister formation and papillary dermal edema. Dermis also showed perivascular and periadnexal mononuclear cell infiltrate and erythrocyte extravasation (Figure 4).

These lesions of SLE with blisters healed within 3 weeks with potent topical steroids. For 7 months after the diagnosis of SLE with blisters, the patient's condition was well controlled with progressively decreasing doses of prednisone and hydroxychloroquine, in the absence of cyclophosphamide. Despite taking a low dose of prednisone (0.25 mg/kg/day), the patient developed a pulmonary complication that was believed to be the result of sepsis. The patient later presented with pulmonary hemorrhage and death. Because all cultures were negative and aspirin had been inadvertently withdrawn several weeks before, it is not clear whether the final complication was due to a massive pulmonary thromboembolism or to septic shock.

## Discussion

Blistering eruptions are rare cutaneous manifestations of SLE that can result from two distinct mechanisms, as illustrated by the two patients described here. In the first case, vesicles resulted from a subepidermal blistering disease with an acute neutrophil-predominant infiltrate in the upper dermis, known as bullous SLE [1,13]. In the second case, blisters developed from hydropic degeneration of the basal layer and severe edema in the upper dermis, also referred to as SLE with blisters [1,14].

Bullous SLE is a rare, transient autoimmune bullous disease that occurs in established cases of SLE [11]. It appears in less than 5% of patients with SLE, either in isolation or in addition to other cutaneous manifestations [15]. This condition usually affects young females, with only eleven cases reported in children [3-13] until now.

The association of bullous SLE with lupus nephritis has been reported in adults [15-17]. It appears that the close relationship between bullous SLE and lupus nephritis is also important in children, as demonstrated by Sirka et al [11], Vijayalakshmi and Jayavardhana [12], Kumar and Agarwal [13], and the first of the two cases presented here.

The relationship between lupus nephritis and bullous SLE does not appear to be casual. Onetti Muda et. al [18] demonstrated that type VII collagen, which is usually not present in normal glomeruli, is actively synthesized and incorporated in areas of glomerular and/or tubular scarring, irrespective of the underlying disease (including SLE nephritis). These findings confirm the *de novo *expression of fibrillary collagens in the diseased renal extracellular matrix.

Systemic lupus erythematosus is a multisystem heterogeneous autoimmune disease and auto-antibodies directed against several components of the cell have been described. It can be hypothesized that bullous SLE develops in patients with lupus nephritis once type VII collagen deposition has occurred in an abnormal location (e.g., the kidney) and antibody production towards this abnormally located protein has been initiated. Type VII collagen is the major component of anchoring fibrils at the dermal-epidermal junction, and is the target of autoimmunity in patients with bullous SLE [19]. There has been at least one report of bullous eruption as the first clinical sign of SLE relapse [20] suggesting that skin damage can be a marker of disease activity.

The second patient described in this report developed SLE with blisters, which is also a rare manifestation of SLE both in adults and children. The actual incidence of this condition is difficult to ascertain, especially because some cases are reported under the term 'bullous SLE', even when the patients do not have the typical autoimmune blistering disease with acute neutrophilic upper dermal infiltrate and subepidermal separation, as well as positive direct immunofluorescence tests. However, of the 148 pediatric SLE cases that have been examined in our lupus clinic over the last 8 years, this is our first incidence of SLE with blisters.

As opposed to bullous SLE, SLE with blisters has not been associated with specific systemic lupus manifestations and therefore does not necessarily imply a worse prognosis. This LE-specific lesion represents a severe dermo-epidermal edema, which is not directed towards specific antigens.

The differential diagnosis of blistering eruptions in patients with SLE includes dermatitis herpetiformis and bullous pemphigoid which are clinically similar but can be differentiated by direct immunofluorescence. Bullous lesions in SLE due to photosensitivity, acute lupus or drugs can be differentiated by both histopathology and immunopathology. Epidermolysis bullosa acquisita (EBA) is histopathologically and immunopathologically identical since both are mediated by circulating antibodies against type VII collagen. However, a dramatic therapeutic response to dapsone in bullous SLE differentiates it from EBA [11]. When in a linear disposition, herpes zoster can be considered in the differential, but it can be easily differentiated by histopathology [6].

It seems contradictory that the patient with bullous SLE continued to thrive, whereas the patient with SLE with blisters, which are apparently not associated with any further risk, had a fatal clinical course. However, the cause of death in the latter case could be related to an APS relapse when the patient developed what has been called "catastrophic APS" after low-dose aspirin was inadvertently withdrawn. There were no other signs or symptoms of SLE disease activity, cyclophosphamide had been stopped, and the prednisone dose was low. Therefore, the patient had a low risk of developing a septic event due to the immunosuppressive effects of medical treatment.

## Conclusions

The recognition and correct diagnosis of two different mechanisms of blistering production in SLE is mandatory, as bullous SLE implies the risk of lupus nephritis and can sometimes be the first manifestation of the disease [16,21], whereas SLE with blisters does not generally imply further risk of disease activity or a bad prognosis.

## Consent

Clinical photographs were taken with oral consent from the parents in both cases. The identity of the patients cannot be disclosed from the reports and the clinical photographs do not reveal any recognizable feature of either. Besides, the Ethical Committee at our Hospital has found no objection to the publication of both cases.

## Competing interests

The authors declare that they have no competing interests.

## Authors' contributions

MSO and FER contributed in taking care of the patients, preparing the manuscript and reviewing the final draft. ELC and BLB contributed in reading the skin biopsies, preparing the manuscript and reviewing the final draft.
